# Convulsive‐Like Movements in Posterior Circulation Large Vessel Occlusion: Associations With Midbrain Injury and Poor Outcome

**DOI:** 10.1002/cns.70980

**Published:** 2026-06-13

**Authors:** Wan Wang, Juntao Yin, Boyi Yuan, Zixin Wang, Jiamin Li, Yun Chen, Jiameng Li, Xinyi Wang, Jiapeng Zhao, Jinming Sheng, Qingfeng Ma

**Affiliations:** ^1^ Department of Neurology Xuanwu Hospital, Capital Medical University Beijing China; ^2^ Department of Neurology Xingtai Central Hospital Xingtai China

**Keywords:** basilar artery occlusion, convulsive‐like movements, midbrain infarction, posterior circulation large vessel occlusion

## Abstract

**Background and Purpose:**

Convulsive‐like movements are an uncommon and frequently misinterpreted manifestation of posterior circulation stroke. Although they are often regarded as non‐epileptic brainstem motor phenomena, their neuroanatomical substrates and prognostic implications in posterior circulation large vessel occlusion (LVO) remain poorly characterized. This study aimed to define the clinical and imaging correlates of convulsive‐like movements and to evaluate their association with functional outcomes.

**Methods:**

We performed a retrospective analysis of prospectively collected data from consecutive adults with posterior circulation LVO admitted between June 2017 and June 2025. Clinical and laboratory data were obtained from a prospectively maintained stroke registry. Neuroimaging was reviewed for ischemic topography and network involvement. Ninety‐day functional outcomes were assessed using the modified Rankin Scale (mRS). Multivariable logistic regression was used to identify imaging features associated with convulsive‐like movements and to assess the relationship between convulsive‐like movements and 90‐day functional outcome. Sensitivity analyses were performed in patients with complete neuroimaging and in the EVT‐treated subgroup.

**Results:**

A total of 269 patients were included (mean age, 62.0 ± 11.8 years; 76.6% male), and convulsive‐like movements occurred in 35 patients (13.0%). Compared with patients without such movements, those with convulsive‐like movements presented with more severe neurological deficits (median NIHSS score, 38 vs. 20; *p* < 0.001) and impaired consciousness (median GCS score, 4 vs. 8; *p* < 0.001). Midbrain infarction was significantly more frequent in patients with convulsive‐like movements (46.4% vs. 19.7%; *p* = 0.001) and remained independently associated with convulsive‐like movements in the imaging‐complete subgroup (adjusted odds ratio [aOR], 3.61; 95% confidence interval [CI], 1.16–11.22; *p* = 0.027). Poor 90‐day functional outcome occurred in 91.4% of patients with convulsive‐like movements, compared with 62.8% of those without (*p* < 0.001). After adjustment for age, baseline NIHSS score, treatment modality, and basilar artery occlusion segment, convulsive‐like movements remained independently associated with poor functional outcome (aOR, 6.38; 95% CI, 1.38–29.44; *p* = 0.018). In sensitivity analyses restricted to patients with complete neuroimaging and to EVT‐treated patients, the direction and magnitude of this association were similar.

**Conclusions:**

In posterior circulation LVO, convulsive‐like movements are an underrecognized but clinically important presentation associated with midbrain injury and unfavorable functional outcome. These findings support improved recognition of this presentation and warrant further prospective validation.

## Graphical abstract

Graphical abstract illustrating the association between convulsive‐like movements, midbrain injury, and unfavorable 90‐day functional outcome in posterior circulation large vessel occlusion (LVO).

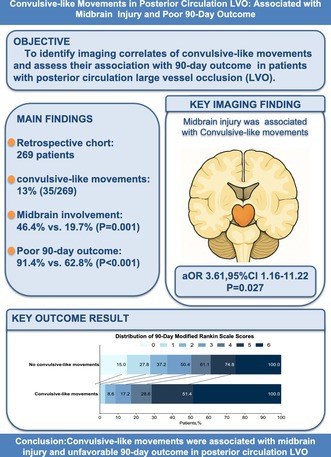

## 1 Introduction

Convulsive seizures are well‐recognized complications of ischemic stroke, occurring in approximately 2%–10% of patients and typically arising from cortical involvement in anterior circulation infarction [1, 2]. In posterior circulation stroke, however, paroxysmal convulsive‐like movements represent an underrecognized and diagnostically challenging manifestation. These brief, stereotyped motor events often mimic epileptic seizures and have been described in basilar artery occlusion and top‐of‐the‐basilar syndromes [3, 4, 5], yet their mechanistic substrate and prognostic significance remain insufficiently defined. In the hyperacute setting, misclassifying these episodes as epileptic seizures may delay vascular imaging, reperfusion therapy, or appropriate airway management.

Posterior circulation large vessel occlusion (LVO), particularly basilar artery occlusion, carries an exceptionally high risk of mortality and disability despite recent advances in endovascular therapy demonstrated in BASICS, ATTENTION, and BAOCHE [6, 7, 8]. Clinical presentation is frequently dominated by impaired consciousness, respiratory compromise, and rapidly evolving neurological deterioration [9]. Whether convulsive‐like movements simply reflect this malignant phenotype or instead identify a distinct brainstem‐driven motor manifestation within posterior circulation LVO remains unknown.

Emerging neurophysiological and imaging evidence suggests that these episodes are unlikely to be epileptic in origin. Instead, they may arise from transient dysfunction of brainstem motor–arousal circuits, including the midbrain reticular formation, thalamic relay nuclei, and descending reticulospinal pathways [10, 11, 12, 13]. The midbrain is increasingly recognized as a critical integrative hub within arousal–motor networks [14, 15], and focal midbrain ischemia may induce seizure‐like motor activity through disruption of dopaminergic modulation and brainstem gating mechanisms [16, 17]. Despite accumulating case‐based observations [3, 4, 5, 12], systematic evaluation of the clinicoradiologic correlates of these events, particularly the role of midbrain involvement, has been lacking. Moreover, their prognostic implications have not been examined in a well‐defined posterior circulation LVO cohort.

To address these uncertainties, we evaluated the clinical profile, imaging correlates with a particular focus on midbrain involvement, and 90‐day functional outcomes associated with convulsive‐like movements in a well‐defined posterior circulation LVO cohort. By clarifying the mechanistic and prognostic relevance of these underrecognized motor events, our study may improve diagnostic accuracy, facilitate timely reperfusion decision‐making, and inform neurocritical care in posterior circulation stroke.

## 2 Methods

### 2.1 Standard Protocol Approvals and Patient Consent

The study was approved by the Ethics Committee of Xuanwu Hospital, Capital Medical University, as a retrospective analysis of a prospectively maintained institutional stroke database. Informed consent was waived because of the observational design and use of deidentified data. The study adhered to the STROBE guidelines.

### 2.2 Study Population

We retrospectively identified consecutive patients with posterior circulation LVO admitted to Xuanwu Hospital between June 2017 and June 2025. Eligible patients were aged 18 years or older, presented within 72 h of symptom onset, had basilar artery or bilateral vertebral artery occlusion confirmed by computed tomography angiography (CTA) or digital subtraction angiography (DSA), and had a premorbid modified Rankin Scale (mRS) score of 2 or lower. Patients were excluded if they had incomplete clinical data, abandoned treatment in the emergency department, were subsequently transferred or discharged home before receiving standard treatment, were lost to 90‐day follow‐up, or developed convulsive‐like movements attributable to nonischemic causes, such as metabolic or toxic abnormalities, drug withdrawal, or preexisting epilepsy.

Convulsive‐like movements were defined as brief, paroxysmal, involuntary motor events occurring during the hyperacute ischemic phase of posterior circulation LVO. Events typically included intermittent jerky or shaking movements, shivering‐like or tremulous movements [13]. To improve exposure ascertainment across care settings, we systematically reviewed all available prehospital and in‐hospital documentation, including prehospital history recorded at presentation and emergency department triage notes. Episodes were considered present only when they were directly observed by medical staff or reliably reported by witnesses or family members and contemporaneously documented in the medical record. The time of first occurrence was defined as the earliest documented convulsive‐like movement across all available records. In all affected patients, the first documented episode occurred before the initiation of deep sedation and/or neuromuscular blockade.

Clinical, laboratory, and neuroimaging data were derived from a prospectively maintained institutional stroke registry and independently verified by 2 investigators blind to clinical outcomes.

### 2.3 Clinical and Laboratory Assessments

Baseline demographics, vascular risk factors, and prestroke mRS were recorded. Neurological severity was assessed on admission using the National Institutes of Health Stroke Scale (NIHSS). Presenting features included dizziness, central hypoventilation, convulsive‐like movements, hyperhidrosis, and impaired consciousness. Motor and language deficits were not systematically assessed because many patients had impaired consciousness and/or required airway support at presentation, making standardized bedside evaluation unreliable in this posterior circulation stroke cohort. Laboratory parameters included white blood cell count and neutrophil count.

### 2.4 Neuroimaging Analysis

Noncontrast CT or MRI was reviewed to determine infarct distribution across posterior circulation territories. Ischemic burden was quantified using the posterior circulation–Alberta Stroke Program Early CT Score (pc‐ASPECTS). In the imaging‐complete subgroup, pc‐ASPECTS was determined on diffusion‐weighted imaging (DWI), and all included patients underwent MRI within 3 days of stroke onset, regardless of treatment strategy. Two experienced readers blind to clinical data independently reviewed the images.

Midbrain involvement was defined as any acute DWI‐positive lesion within the midbrain (mesencephalon), based on the arterial territory framework described by Tatu et al. [18], and the DWI‐based brainstem lesion approach proposed by Cho et al. [19], A representative example is provided in Figure S1. We adapted the PMT (pons–midbrain–thalamus) framework described by Liu et al. [20], to capture lesions affecting arousal and motor integration networks. In parallel, we defined a temporo‐occipital (TO) network based on topographic patterns described in posterior circulation stroke syndromes [21]. Deep‐cortical network involvement was categorized as combined PMT–TO involvement, corresponding to disruption of subcortical and higher‐order association circuits.

The basilar artery occlusion segment (proximal, middle, or distal) was ascertained for all patients from operative records, CTA findings, or angiographic report descriptions. Among the 269 enrolled patients, 251 (28 with and 223 without convulsive‐like movements) had complete MRI data.

### 2.5 Reperfusion Therapy, Complications, and Functional Outcomes

Patients received medical therapy alone, intravenous thrombolysis (IVT), and/or endovascular thrombectomy (EVT) according to contemporary guidelines and individual treatment eligibility. For patients undergoing endovascular treatment, door‐to‐puncture time and onset‐to‐reperfusion time were recorded. Successful reperfusion was defined as a modified Thrombolysis in Cerebral Infarction (mTICI) score of 2b or 3.

In‐hospital complications, including pneumonia, urinary tract infection, deep vein thrombosis, stress ulcer, hypoproteinemia, hepatic or renal dysfunction, cardiovascular complications, and cerebral herniation, were documented. The need for mechanical ventilation, neuro‐intensive care unit (NICU) admission, and NICU length of stay were recorded.

Functional outcome at 90 days was assessed using the mRS by a blind assessor with a structured interview conducted either face‐to‐face or by telephone. Poor outcome was defined as an mRS score of 3 to 6. Mortality was assessed both in the hospital and at 90 days.

### 2.6 Statistical Analysis

Continuous variables were summarized as mean (SD) or median (IQR), as appropriate, and compared using the independent‐samples *t*‐test or Mann–Whitney U test. Categorical variables were compared using the χ^2^ test or Fisher's exact test, as appropriate.

Given the limited number of patients with convulsive‐like movements, the multivariable imaging analysis was performed using a prespecified parsimonious logistic regression model including midbrain involvement, TO involvement, and pc‐ASPECTS. These variables were selected a priori on the basis of clinical relevance and pathophysiologic plausibility rather than by stepwise variable selection.

A separate multivariable logistic regression model was constructed to evaluate factors associated with poor 90‐day functional outcome (mRS 3–6) in the full cohort (*n* = 269). This model included a prespecified parsimonious set of clinically relevant covariates selected a priori rather than through stepwise procedures. Covariates included convulsive‐like movements, age, baseline NIHSS score, treatment modality (medical therapy only, IVT only, and EVT‐based therapy), and basilar artery occlusion segment (proximal, middle, and distal).

In patients undergoing EVT, a secondary EVT‐restricted multivariable logistic regression analysis was performed with further adjustment for reperfusion success defined by mTICI grade (2b‐3 vs. 0‐2a). Adjusted odds ratios (aORs) and 95% confidence intervals (CIs) were reported. Model calibration was assessed using the Hosmer–Lemeshow test. All tests were two‐sided, and *p* < 0.05 was considered statistically significant. Analyses were performed using SPSS version 26.0 (IBM Corp) and R version 4.2.2.

### 2.7 Sensitivity Analysis

A prespecified sensitivity analysis was conducted in the imaging‐complete subgroup (*n* = 251) to assess whether the association between convulsive‐like movements and poor 90‐day functional outcome remained robust after restricting the analysis to patients with complete neuroimaging data. Using the same covariates as in the primary full‐cohort outcome model, the sensitivity model additionally included pc‐ASPECTS to account for overall posterior circulation ischemic burden.

## 3 Results

### 3.1 Baseline Characteristics, Clinical Manifestations, and Laboratory Findings

**FIGURE 1:**
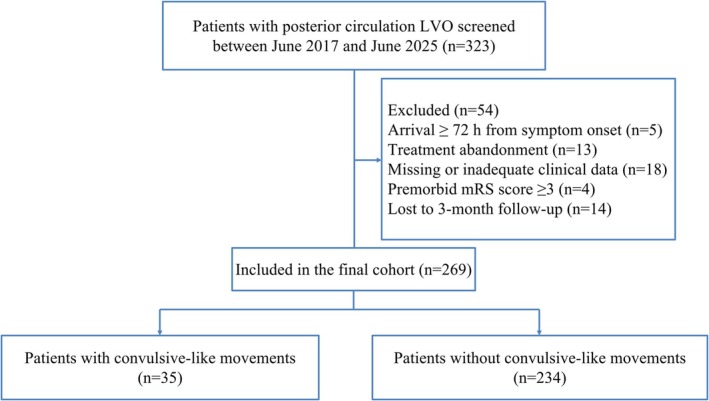
Patient selection flow diagram. AIS, acute ischemic stroke; LVO, large vessel occlusion; mRS, modified Rankin Scale.

Among the 323 patients screened, 269 (83.3%) met the inclusion criteria and were enrolled in the final cohort (mean age, 62.0 ± 11.8 years; 76.6% male; Figure 1). Convulsive‐like movements were observed in 35 patients (13.0%). As shown in Table 1, demographic characteristics were largely comparable between patients with and without convulsive‐like movements. Age, sex, and the prevalence of major vascular risk factors, including hypertension, diabetes mellitus, atrial fibrillation, dyslipidemia, coronary artery disease, and prior antithrombotic therapy, did not differ significantly between groups (all *p* > 0.05). Prestroke functional status was also similar, with a high proportion of patients in both groups having a premorbid mRS score of 0 (91.4% vs. 86.8%; *p* = 0.61).

**TABLE 1:** Demographics, clinical manifestations, and laboratory test results of patients with large vessel occlusion in the posterior circulation.

	Convulsive‐like movements (*n* = 35)	No convulsive‐like movements (*n* = 234)	*p*
Age, years, mean (SD)	59.9 (13.9)	62.1 (11.4)	0.310
Male, *n* (%)	31 (88.6%)	175 (74.8)	0.072
Medical history and medication history, *n* (%)			
Previous ischemic stroke	7 (20)	58 (24.8)	0.537
Hypertension	25 (71.4)	175 (74.8)	0.671
Diabetes mellitus	6 (17.1)	59 (25.2)	0.298
Hyperlipidemia	12 (34.3)	79 (33.8)	0.951
Atrial fibrillation	4 (11.4)	41 (17.5)	0.368
Coronary atherosclerotic heart disease	4 (11.4)	42 (17.9)	0.339
Previous antiplatelet therapy	5 (14.3)	35 (15)	0.732
Previous anticoagulation therapy	0 (0)	11 (4.7)	0.394
Smoking history	12 (35)	108 (46.2)	0.188
Drinking history	14 (40)	95 (40.6)	0.964
mRS score of 0 before stroke, *n* (%)	32 (91.4)	203 (86.8)	0.614
GCS, median (IQR)	4 (3–6)	8 (4–13)	< 0.001
NIHSS, median (IQR)	38 (23–40)	20 (11–31)	< 0.001
Clinical manifestations, *n* (%)			
Dizziness	16 (45.7)	102 (43.6)	0.813
Central hypoventilation	1 (2.9)	3 (1.3)	0.429
Hyperhidrosis	4 (11.4)	6 (2.6)	0.035
Impaired consciousness	29 (82.9)	120 (51.3)	< 0.001
Laboratory parameters			
White blood cell count, median (IQR), ×10^9^/L	13.15 (9.8–16.27)	10.14 (8.06–12.66)	< 0.001
Neutrophil count, median (IQR), ×10^9^/L	11.51 (7.98–14.42)	8.27 (6.35–10.39)	< 0.001

Abbreviations: GCS, Glasgow Coma Scale; mRS, modified Rankin Scale; NIHSS, National Institutes of Health Stroke Scale.

Despite these broadly similar baseline characteristics, patients with convulsive‐like movements had substantially more severe neurological impairment at presentation. Median NIHSS score was markedly higher in patients with convulsive‐like movements than in those without (38 vs. 20; *p* < 0.001), whereas median GCS score was substantially lower (4 vs. 8; *p* < 0.001). Clinical manifestations were consistent with this greater severity: Impaired consciousness was more frequent in the convulsive‐like movement group (82.9% vs. 51.3%; *p* < 0.001), and hyperhidrosis, a possible autonomic marker, was also more common (11.4% vs. 2.6%; *p* = 0.035). Other presenting features, including dizziness and central hypoventilation, did not differ significantly between groups.

Laboratory findings further suggested greater systemic inflammatory activation in patients with convulsive‐like movements. White blood cell count and neutrophil count were both significantly higher in this group (both *p* < 0.001). In contrast, lymphocyte, monocyte, and platelet counts did not differ significantly between groups (not shown).

Overall, these findings indicate that convulsive‐like movements were associated with greater initial neurological severity, impaired consciousness, and elevated systemic inflammatory markers, despite similar demographic characteristics and vascular risk profiles.

### 3.2 Imaging Characteristics

Neuroimaging data were available for 28 of 35 patients with convulsive‐like movements (80.0%) and 223 of 234 without such movements (95.3%). The distribution of infarction across posterior circulation structures differed between groups in several key regions.

Midbrain infarction was significantly more frequent in patients with convulsive‐like movements than in those without such movements (46.4% vs. 19.7%; *p* = 0.001), representing the most prominent structural difference between groups. Occipital lobe involvement was also more common in the convulsive‐like group (60.7% vs. 39.5%; *p* = 0.032), suggesting concurrent disruption of visual association or thalamo‐occipital pathways. At the network level, combined PMT‐TO network involvement occurred in nearly two‐thirds of patients with convulsive‐like movements (64.3% vs. 35.9%; *p* = 0.004), whereas isolated PMT or isolated TO involvement did not differ significantly between groups.

Ischemic burden assessed using pc‐ASPECTS was greater in patients with convulsive‐like movements (median 5 [IQR 3–6] vs. 6 [IQR 5–8]; *p* = 0.015), indicating more extensive posterior circulation injury. Despite this heavier ischemic burden, rates of any intracranial hemorrhage (32.1% vs. 21.1%; *p* = 0.185) and symptomatic hemorrhage (14.3% vs. 10.3%; *p* = 0.752) were comparable between groups, as detailed in Table S1.

**FIGURE 2:**
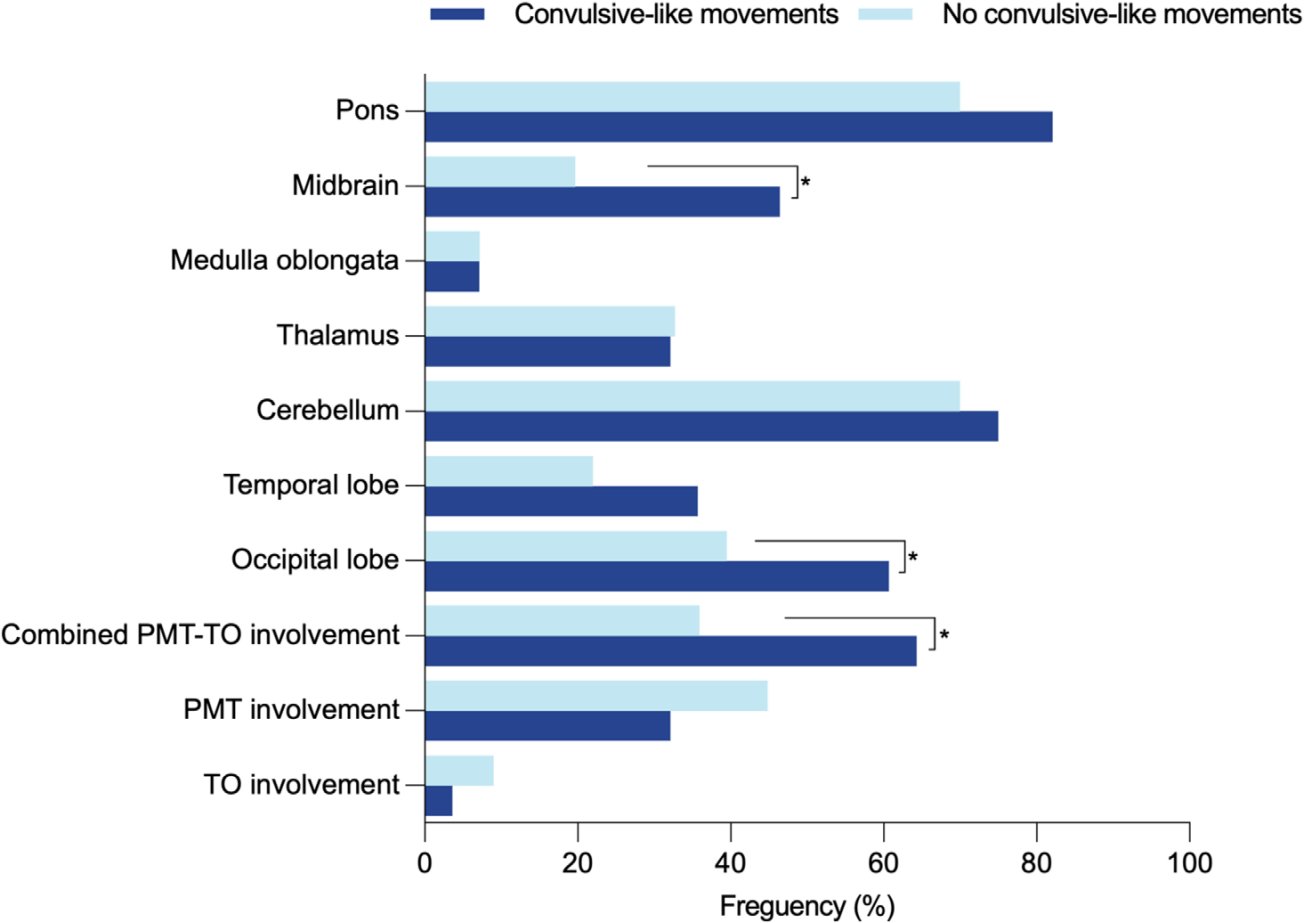
Neuroimaging Features in Patients With and Without Convulsive‐Like Movements. Bars represent the proportions of patients with each neuroimaging feature in the 2 groups. * indicates nominal between‐group differences at *p* < 0.05. PMT‐TO, combined involvement of the pons–midbrain–thalamus and temporo‐occipital regions; PMT, pons–midbrain–thalamus regions; TO, temporo‐occipital regions.

These neuroimaging findings are illustrated in Figure 2, with additional imaging details provided in Table S1, which highlights the higher frequencies of midbrain infarction, occipital lobe infarction, and combined PMT–TO network involvement in patients with convulsive‐like movements. Together, these findings support a distinct brainstem–cortical injury pattern associated with convulsive‐like movements.

### 3.3 Treatment, Complications, and Functional Outcomes

Acute reperfusion therapies were used at similar rates in the 2 groups. Intravenous thrombolysis was administered in 20.0% of patients with convulsive‐like movements and 20.9% of those without such movements (*p* = 0.90), and the frequency of EVT was likewise comparable (85.7% vs. 86.3%; *p* = 1.00). Among patients undergoing EVT, onset‐to‐revascularization time was significantly shorter in the convulsive‐like movements group (median, 477 vs. 590 min; *p* = 0.045), whereas door‐to‐groin puncture time did not differ significantly between groups (153.5 vs. 140 min). This pattern suggests that the shorter overall reperfusion time was more likely related to earlier prehospital recognition and triage in patients with more severe initial presentations than to differences in in‐hospital workflow.

Complication profiles differed substantially between groups. Patients with convulsive‐like movements had a higher incidence of cerebral herniation (20.0% vs. 6.4%; *p* = 0.016), and the need for mechanical ventilation was markedly more frequent (88.6% vs. 39.7%; *p* < 0.001). Consistent with this greater illness severity, NICU admission was more common (82.9% vs. 47.9%; *p* < 0.001), and NICU stay was longer (median, 4 vs. 0 days; *p* = 0.003). Rates of systemic complications, including pneumonia, urinary tract infection, deep vein thrombosis, stress ulcer, and organ dysfunction, were generally higher in patients with convulsive‐like movements, although not all differences reached statistical significance. Mortality was also higher in this group: In‐hospital mortality was 28.6% vs. 14.1% (*p* = 0.029), and 90‐day mortality was 48.6% vs. 25.2% (*p* = 0.004) (Table 2).

**TABLE 2:** Management, Complications, and Short‐Term Outcomes in Patients with Posterior Circulation Large Vessel Occlusion.

	Convulsive‐like movements (*n* = 35)	No convulsive‐like movements (*n* = 234)	*p*‐value
Reperfusion therapy			
Intravenous thrombolysis, *n* (%)	7 (20)	49 (20.9)	0.898
Mechanical thrombectomy, *n* (%)	30 (85.7)	202 (86.3)	1.0
Median duration, median (IQR), min			
From door to groin puncture	153.5 (107–208)	140 (116–184)	0.715
From stroke onset to revascularization	477 (340–834)	590 (442–955)	0.045
Complication, *n* (%)			
New cardiovascular events	6 (17.1)	38 (16.2)	0.893
Pneumonia	30 (85.7)	175 (74.8)	0.157
Urinary tract infection	3 (8.6)	22 (9.4)	1.0
Deep vein thrombosis	5 (14.3)	50 (21.4)	0.333
Stress ulcer	9 (25.7)	49 (20.9)	0.522
Hypoproteinemia	19 (54.3)	110 (47.0)	0.422
Liver dysfunction	18 (51.4%)	84 (35.9)	0.077
Renal dysfunction	6 (17.1)	29 (12.4)	0.61
Cerebral herniation	7 (20)	15 (6.4%)	0.016
Clinical outcome			
Mechanical ventilation, *n* (%)	31 (88.6)	93 (39.7)	< 0.001
NICU admission, *n* (%)	29 (82.9)	112 (47.9)	< 0.001
Length of NICU stay, median (IQR), d	4 (1–18)	0 (0–10)	0.003
In‐hospital mortality, *n* (%)	10 (28.6)	33 (14.1)	0.029
Death within 90 d, *n* (%)	17 (48.6)	59 (25.2)	0.004

Abbreviation: NICU, neurologic intensive care units.

**FIGURE 3:**
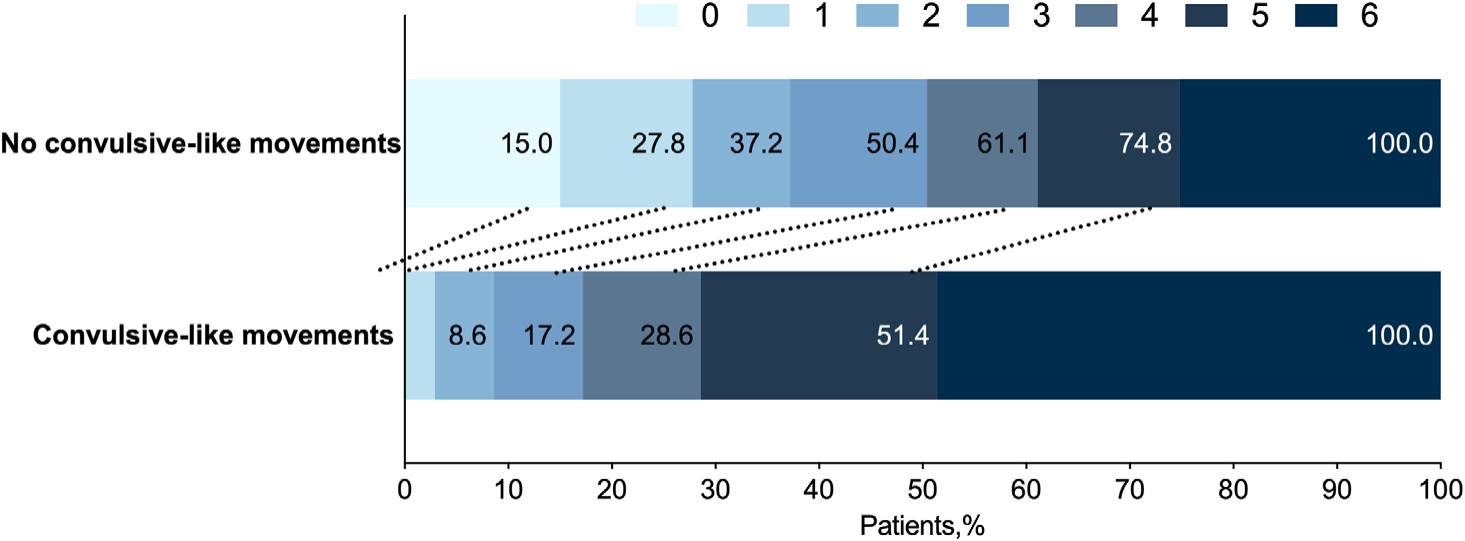
Distribution of 90‐Day Modified Rankin Scale Scores.

Functional outcomes were markedly worse in patients with convulsive‐like movements. A clearly rightward shift in the distribution of mRS was observed in this group (Figure 3), and poor 90‐day functional outcome (mRS score 3–6) occurred in 91.4% of patients with convulsive‐like movements compared with 62.8% of those without (*p* < 0.001).

### 3.4 Multivariable Analyses

**FIGURE 4:**
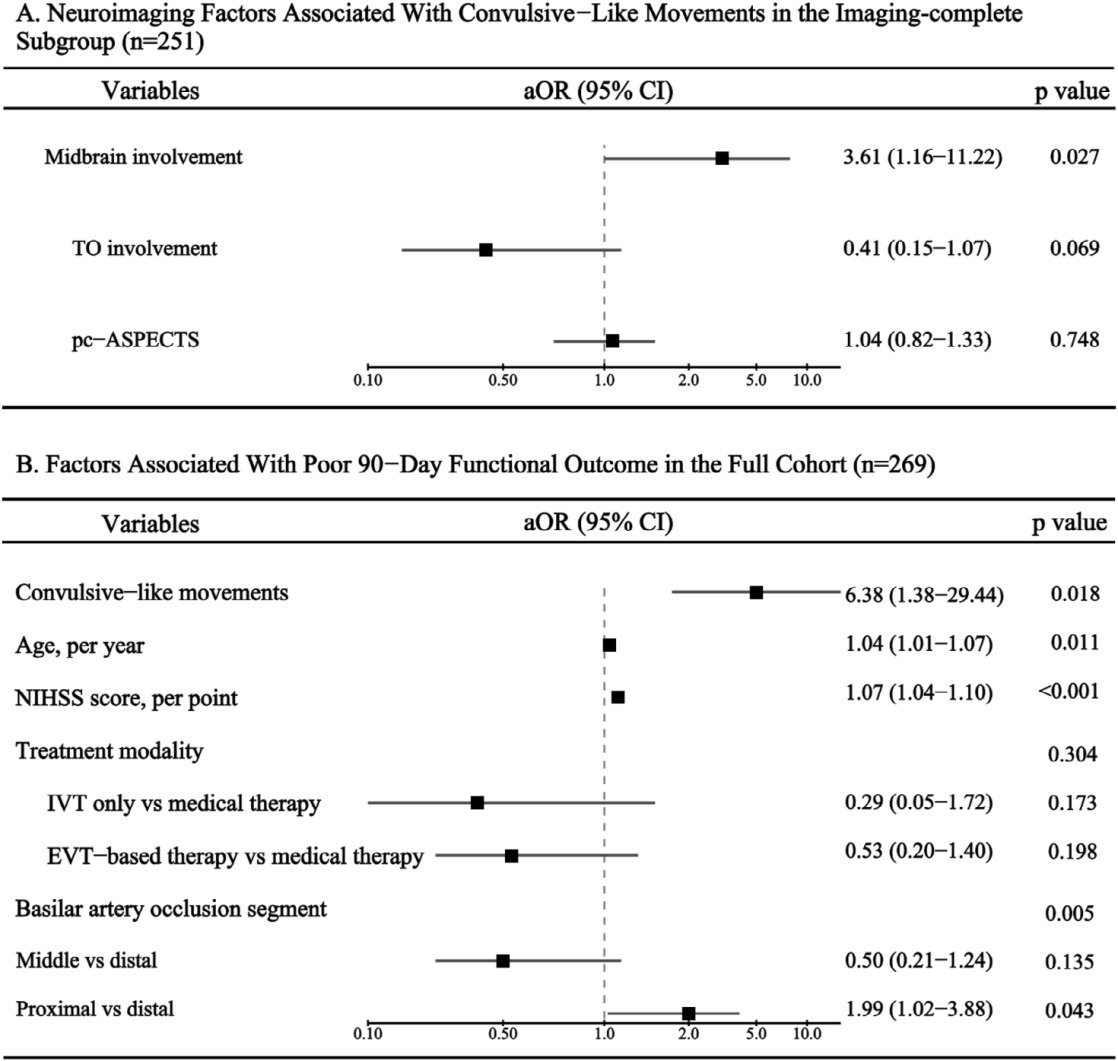
Factors Associated With Convulsive‐Like Movements and Poor 90‐Day Functional Outcomes. (A) Associations between neuroimaging factors and convulsive‐like movements in the imaging‐complete subgroup of patients with posterior circulation large vessel occlusion. (B) Associations between clinical and treatment variables and poor 90‐day functional outcome (modified Rankin Scale score 3–6) in the full cohort after multivariable adjustment. Error bars indicate 95% confidence intervals. AOR, adjusted odds ratio; CI, confidence interval; EVT, endovascular thrombectomy; IVT, intravenous thrombolysis; NIHSS, National Institutes of Health Stroke Scale; pc‐ASPECTS, posterior circulation Alberta Stroke Program Early CT Score; TO, temporo‐occipital regions.

In the imaging‐based multivariable model including patients with complete neuroimaging data (*n* = 251), midbrain involvement remained independently associated with convulsive‐like movements (aOR, 3.61; 95% CI, 1.16–11.22; *p* = 0.027), whereas TO involvement and pc‐ASPECTS were not independently associated (Figure 4A).

In the multivariable model for 90‐day functional outcome in the full cohort (*n* = 269), convulsive‐like movements remained independently associated with poor 90‐day outcome (aOR, 6.38; 95% CI, 1.38–29.44; *p* = 0.018), after adjustment for age, baseline NIHSS score, treatment modality, and basilar artery occlusion segment. Older age (aOR, 1.04 per year; 95% CI, 1.01–1.07; *p* = 0.011), higher baseline NIHSS score (aOR, 1.07 per point; 95% CI, 1.04–1.10; *p* < 0.001), and proximal vs. distal basilar artery occlusion (aOR, 1.99; 95% CI, 1.02–3.88; *p* = 0.043) were also independently associated with poor outcome, whereas treatment modality was not independently associated with poor outcome (Figure 4B).

In a secondary EVT‐restricted multivariable model with additional adjustment for reperfusion success defined by mTICI grade, convulsive‐like movements remained independently associated with poor 90‐day outcome (aOR, 11.29; 95% CI, 1.41–90.70; *p* = 0.023). Other associations were broadly similar to those in the main model, as shown in Figure S2.

### 3.5 Sensitivity Analysis

**FIGURE 5:**
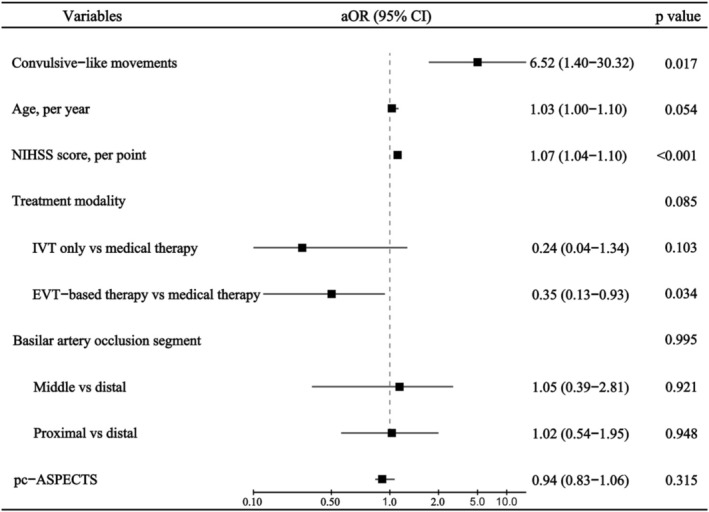
Sensitivity Analysis of Factors Associated With Poor 90‐Day Functional Outcome in the Imaging‐complete Subgroup (*n* = 251). AOR, adjusted odds ratio; CI, confidence interval; EVT, endovascular thrombectomy; IVT, intravenous thrombolysis; NIHSS, National Institutes of Health Stroke Scale; pc‐ASPECTS, posterior circulation Alberta Stroke Program Early CT Score.

Sensitivity analyses restricted to the imaging‐complete subgroup (*n* = 251) were consistent with the primary outcome model. After additional adjustment for pc‐ASPECTS, convulsive‐like movements remained independently associated with poor 90‐day functional outcome (aOR, 6.52; 95% CI, 1.40–30.32; *p* = 0.017). Baseline NIHSS score also remained independently associated with poor outcome, whereas pc‐ASPECTS was not. Treatment modality was not significant overall, although EVT‐based therapy vs. medical therapy reached significance in one category‐specific comparison (Figure 5). Overall, these findings support the robustness of the primary outcome analysis.

## 4 Discussion

In this cohort of posterior circulation LVO, convulsive‐like movements occurred in approximately 13% of patients and were strongly associated with midbrain infarction, greater clinical severity, and markedly unfavorable 90‐day outcomes. Even after adjustment for age, baseline neurological severity, treatment modality, and basilar artery segment, convulsive‐like movements remained independently associated with poor functional outcome. These findings suggest that convulsive‐like movements may represent a recognizable clinical presentation associated with severe brainstem injury, rather than merely reflecting overall ischemic burden alone. To our knowledge, this study provides the first systematic cohort‐level characterization of convulsive‐like movements in posterior circulation LVO, integrating clinical presentation, inflammatory markers, and network‐based neuroimaging features.

These episodes appear clinically distinct from typical poststroke seizures, which usually arise from cortical injury and are more commonly observed in anterior circulation infarction [11, 12]. Previous reports in posterior circulation stroke have largely been limited to isolated cases or small case series, often emphasizing diagnostic uncertainty without systematic neuroanatomical validation [3, 4, 5, 13]. Our findings extend this literature by demonstrating a strong association between convulsive‐like movements and midbrain infarction, consistent with involvement of brainstem arousal–motor circuits [13].

The observed association with midbrain injury provides a plausible mechanistic framework, although any mechanistic interpretation remains inferential. The midbrain contains key components of the ascending reticular activating system, dopaminergic midbrain nuclei, the periaqueductal gray, the mesencephalic locomotor region, and descending reticulospinal pathways [10, 14, 15, 17]. These structures contribute to consciousness, arousal–motor coupling, autonomic regulation, and patterned motor output. Ischemic dysfunction within these hubs may produce transient tonic or tremulous motor phenomena through disinhibition of subcortical motor generators, in line with both early brainstem stimulation experiments and contemporary neurophysiologic models [16, 22]. Disruption of thalamo‐cortical networks may further contribute to pathological synchronization, impaired arousal transitions, and motor dysregulation, as suggested by modern connectomic studies and ultra‐high‐field functional imaging of brainstem nuclei [14, 15, 17]. However, these interpretations remain inferential because systematic EEG and synchronized video documentation were not available to distinguish non‐epileptic brainstem motor phenomena from epileptic seizures or mixed mechanisms. In addition, the higher frequency of occipital infarction in affected patients raises the possibility of cortical contribution in a subset of cases. The association with hyperhidrosis is also consistent with involvement of dorsal midbrain sympathetic centers and arousal‐related autonomic relays [12].

The motor–autonomic profile of these episodes overlaps in part with paroxysmal sympathetic hyperactivity (PSH), which has been described in brainstem injury, traumatic brain injury, and basilar artery occlusion [23, 24, 25]. Although overt PSH was not observed in our cohort, both conditions may reflect transient disinhibition within brainstem arousal–motor–autonomic circuits. The higher leukocyte and neutrophil counts observed in affected patients may indicate a more intense systemic stress response or heightened inflammatory activation [26], although this interpretation remains speculative.

The clinical importance of early recognition is substantial. Contrary to the concerns that convulsive‐like movements might delay diagnosis, the onset‐to‐reperfusion time was shorter in affected patients. Given that door‐to‐puncture time was similar between groups, the shorter overall reperfusion time likely reflects earlier prehospital prioritization triggered by profound coma, respiratory compromise, or striking motor manifestations rather than differences in in‐hospital workflow. This observation applies to EVT‐treated patients and does not exclude the possibility of misdiagnosis in less experienced settings, where these movements may still be mistaken for epileptic seizures. Recognizing their characteristic clinical pattern—brief generalized tonic or tremulous movements, impaired awareness, absence of clear cortical semiology, and severe consciousness disturbance—should prompt immediate vascular imaging, particularly in patients with suspected posterior circulation stroke. Given the higher frequency of cerebral herniation, mechanical ventilation, and early deterioration in affected patients, early airway protection and close neurocritical monitoring are essential.

Sensitivity analyses in the imaging‐complete subgroup reproduced the main findings, suggesting that the association between convulsive‐like movements and poor outcome was unlikely to be fully explained by missing neuroimaging data. Similarly, in the EVT‐restricted model, the association persisted after additional adjustment for reperfusion success, although the estimate remained imprecise because of the limited sample size. Together with the sensitivity analyses, these findings suggest that the association is not fully explained by conventional severity markers alone, while residual confounding remains possible. Importantly, deeply comatose patients were not excluded, allowing the cohort to capture the malignant clinical spectrum of basilar artery occlusion reported in contemporary EVT trials [6, 7, 8].

## 5 Limitations

This study has several limitations. First, its retrospective single‐center design may introduce selection bias and limit generalizability. Second, the number of patients with convulsive‐like movements was modest, which reduced statistical power for subgroup analyses. Third, neuroimaging was obtained using heterogeneous modalities and protocols, which may have affected lesion characterization despite standardized blind reviewed. Fourth, routine or continuous EEG monitoring and synchronized video documentation were not systematically available. As a result, epileptic seizures could not be reliably distinguished from non‐epileptic brainstem motor phenomena, and mixed mechanisms cannot be excluded. The higher frequency of cortical involvement in affected patients further supports cautious interpretation of the underlying mechanism. Fifth, residual confounding remains possible in the outcome analyses. Some factors relevant to prognosis in basilar artery occlusion, including collateral status and care‐limitation or withdrawal decisions, could not be fully standardized or incorporated across the cohort. In addition, over the long study period, part of the original CTA and angiographic source imaging was no longer available for centralized re‐review, which precluded standardized collateral grading across all patients. Finally, advanced structural and functional connectivity analyses, such as diffusion tractography and resting‐state functional MRI, were not available, precluding more precise mapping of brainstem–cortical network disruption. Future prospective multicenter studies incorporating standardized imaging, electrophysiological monitoring, and high‐resolution connectomic methods are needed to validate these findings and further clarify the mechanisms underlying these events.

## 6 Conclusions

Convulsive‐like movements are an underrecognized but clinically important presentation in posterior circulation LVO, associated with midbrain injury and unfavorable functional outcome. They may serve primarily as a marker of severe brainstem injury and should prompt urgent evaluation and close neurocritical monitoring.

## Funding

This work was supported by Capital's Funds for Health Improvement and Research (CFH) (2022‐2‐3032), the Noncommunicable Chronic Diseases–National Science and Technology Major Project (2024ZD0527600, 2024ZD0527603), and National Key Research and Development Program of China (2023YFC2506504).

## Ethics Statement

This study was approved by the Ethics Committee of Xuanwu Hospital, Capital Medical University (No. Clinical Research [2017] 030).

## Consent

Informed consent was not required due to the observational design and use of deidentified data.

## Conflicts of Interest

The authors declare no conflicts of interest.

## Supporting information


**Figure S1:** Representative axial DWI and corresponding ADC maps illustrating midbrain involvement in a patient with convulsive‐like movements.
**Figure S2:** EVT‐restricted multivariable model for poor 90‐day functional outcome (*n* = 231).
**Table S1:** Imaging characteristics of patients with posterior circulation large vessel occlusion.

## Data Availability

Data are available upon reasonable request to the corresponding author.
