# Supplementary material for: Convulsive‐Like Movements in Posterior Circulation Large Vessel Occlusion: Associations With Midbrain Injury and Poor Outcome
Source: CNS Neurosci Ther. 2026 Jun 13;32(6):e70980. doi: 10.1002/cns.70980 (PMC13263788; doi:10.1002/cns.70980)
Supplement: Supplementary file 1 — Figure S1: Representative axial DWI and corresponding ADC maps illustrating midbrain involvement in a patient with convulsive‐like movements. Figure S2: EVT‐restricted multivariable model for poor 90‐day functional outcome (n = 231). Table S1: Imaging characteristics of patients with posterior circulation large vessel occlusion. [file CNS-32-e70980-s001.docx]

**Supplementary Figure 1. Representative Axial DWI and Corresponding ADC Images Illustrating Midbrain Involvement**


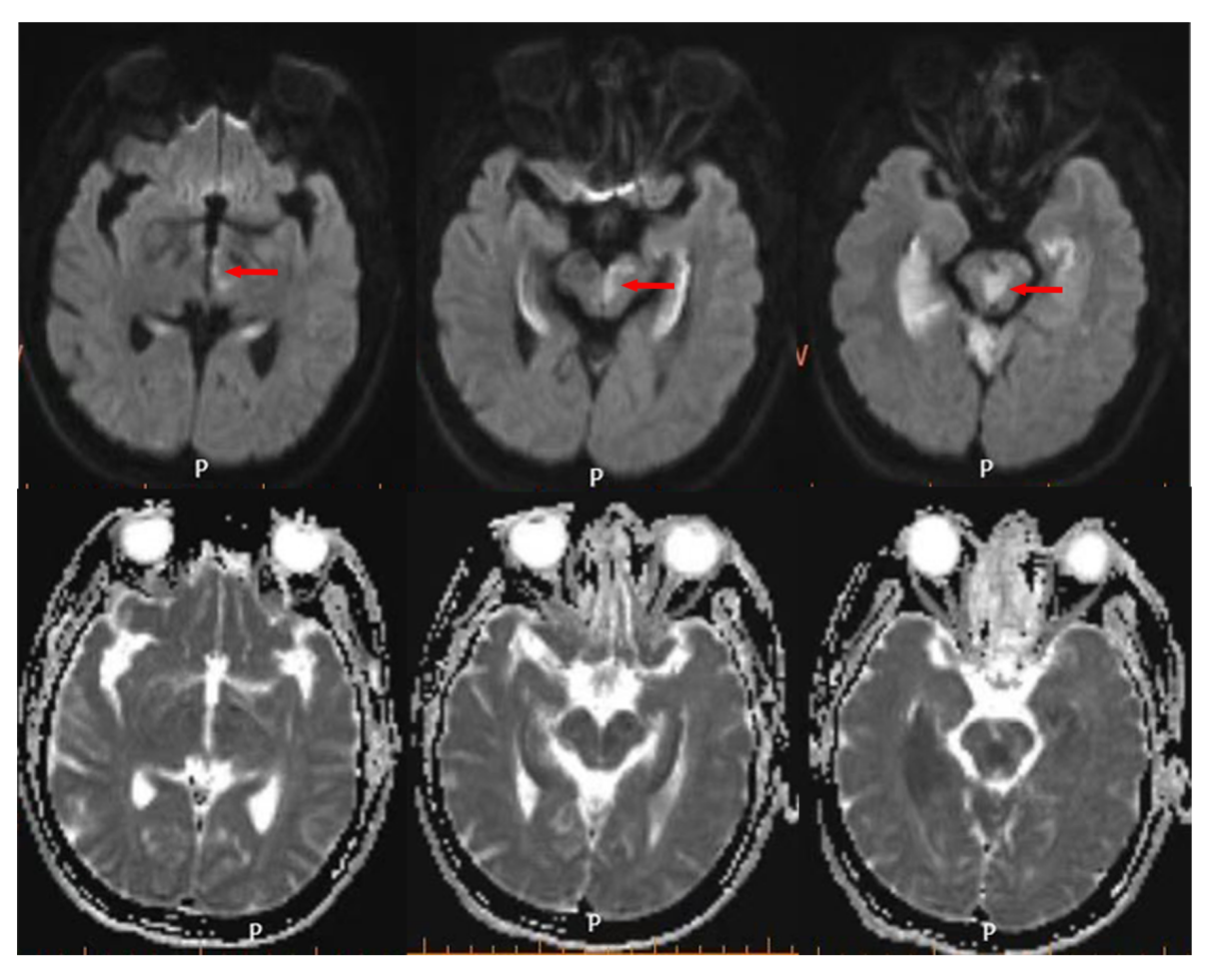


Axial diffusion-weighted imaging and corresponding apparent diffusion coefficient maps from a patient with convulsive-like movements demonstrates acute infarction involving the midbrain on adjacent slices (arrows), These image provide a representative example of the radiologic definition of midbrain involvement used in the present study.

**Supplementary Table 1. Image features of patients with large vessel occlusion in the posterior circulation**

|  | **Convulsive-like group (n=28)** | **No Convulsive-like group(n=223)** | ***p* value** |
| --- | --- | --- | --- |
| Infarction site |  |  |  |
| Pons, n (%) | 23 (82.1) | 156(70) | 0.179 |
| Midbrain, n (%) | 13 (46.4) | 44 (19.7) | 0.001 |
| Medulla oblongata, n (%) | 2 (7.1) | 16 (7.2) | 1.000 |
| Thalamus, n (%) | 9 (32.1) | 73 (32.7) | 0.95 |
| Cerebellum, n (%) | 21 (75) | 156(70) | 0.581 |
| Temporal lobe, n (%) | 10 (35.7) | 49 (22.0) | 0.106 |
| Occipital lobe, n (%) | 17 (60.7) | 88 (39.5) | 0.032 |
| Combined PMT-TO involvement, n (%) | 18 (64.3) | 80 (35.9) | 0.004 |
| PMT involvement, n (%) | 9 (32.1) | 100(44.8) | 0.201 |
| TO involvement, n (%) | 1 (3.6) | 20 (9) | 0.542 |
| pc-ASPECTS, median (IQR) | 5 (3-6) | 6 (5-8) | 0.015 |
| intracranial hemorrhage, n (%) | 9(32.1) | 47 (21.1) | 0.185 |
| Symptomatic intracranial hemorrhage, n (%) | 4 (14.3) | 23 (10.3) | 0.752 |

Abbreviations: PMT, pons-midbrain-thalumus; TO, tempero-occipital; mRS, modified Rankin Scale; pc-ASPECTS, posterior circulation Acute Stroke Prognosis Early CT Score.

**Supplementary Figure 2. EVT-Restricted Multivariable Model for Poor 90-Day Functional Outcome (n=231)**

**
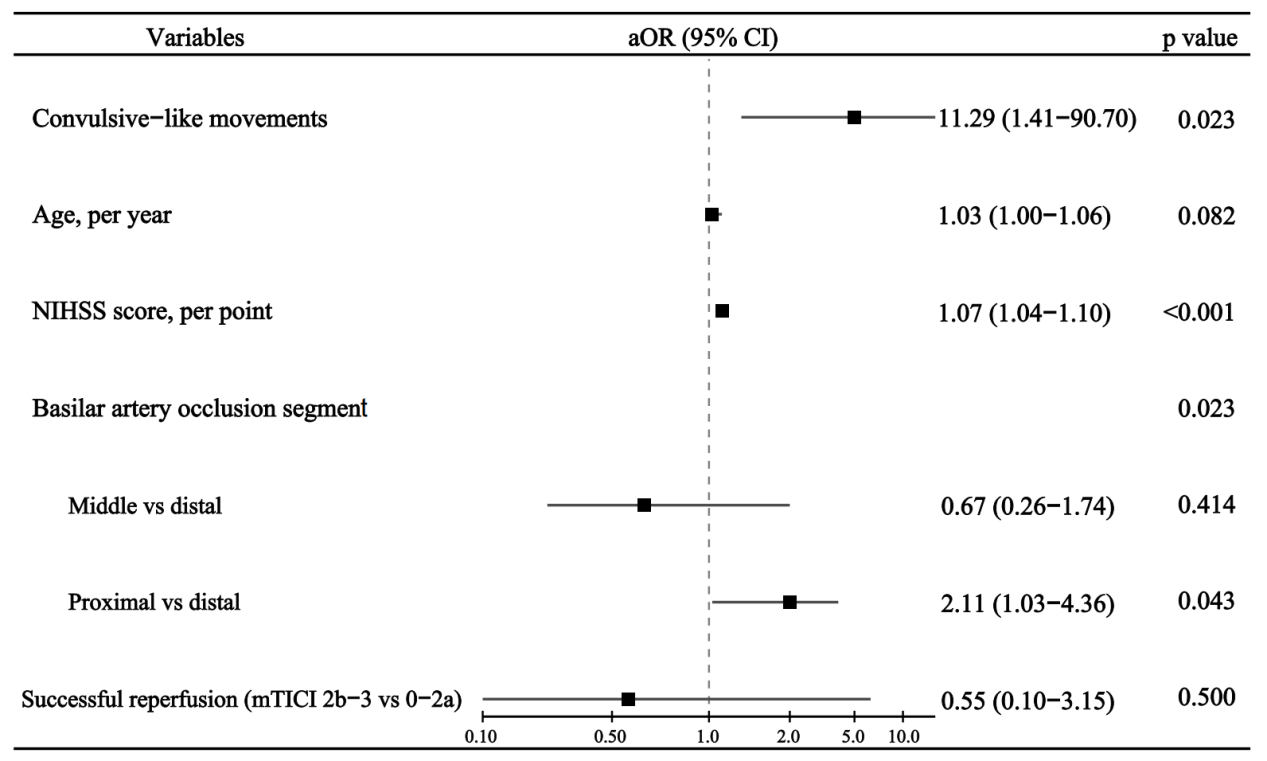
**

Abbreviations: EVT, endovascular thrombectomy; aOR, adjusted odds ratio; CI, confidence interval; NIHSS: National institutes of Health Stroke Scale; mTIC, modified Thrombolysis in Cerebral Infarction.
